# Sulforaphane as a Therapeutic Target for Sarcopenia in Type 2 Diabetes: Mechanistic Insights and Translational Evidence

**DOI:** 10.3390/antiox15101272

**Published:** 2026-10-02

**Authors:** Ayanda Ngcobo, Samukelisiwe C. Shabalala, Nokulunga Hlengwa, Ndivhuwo Muvhulawa, Patrick Orlando, Sonia Silvestri, Luca Tiano, Phiwayinkosi V. Dludla

**Affiliations:** 1 Department of Biochemistry and Microbiology, University of Zululand, KwaDlangezwa 3886, South Africa; shabalalasc@unizulu.ac.za (S.C.S.); hlengwan@unizulu.ac.za (N.H.); 2 Department of Biochemistry, North-West University, Mafikeng Campus, Mmabatho 2735, South Africa; 32917392@mynwu.ac.za; 3 Department of Life and Environmental Sciences, Polytechnic University of Marche, 60131 Ancona, Italy; p.orlando@univpm.it (P.O.); s.silvestri@univpm.it (S.S.); l.tiano@staff.univpm.it (L.T.); 4 School of Medicine, University of KwaZulu-Natal, Durban 4000, South Africa

**Keywords:** sulforaphane, type 2 diabetes, sarcopenia, skeletal muscle, Nrf2 signaling, oxidative stress, inflammation, mitochondrial function, antioxidant therapy, muscle preservation

## Abstract

Sulforaphane, a bioactive isothiocyanate abundant in cruciferous vegetables, has emerged as a potential multi-target therapeutic agent capable of mitigating these pathological processes. Here, using evidence from preclinical and clinical literature, we review the mechanistic and translational evidence linking sulforaphane with the preservation of skeletal muscle tissue in the context of sarcopenia associated with T2D. The reviewed evidence suggests sulforaphane modulates oxidative stress, inflammation, and mitochondrial dysfunction through activation of nuclear factor erythroid 2-related factor 2 (Nrf2) and related signaling pathways. Preclinical rodent studies corroborate these findings, reporting improved muscle mass, contractility, metabolic homeostasis, and attenuation of inflammation via Nrf2/heme oxygenase (HO)-1, AMP-activated protein kinase (AMPK), and peroxisome proliferator-activated receptor-γ coactivator-1α (PGC1α) pathways. Limited clinical evidence from two small studies in healthy adults suggest that short-term sulforaphane supplementation (30 mg/day) may reduce exercise-induced muscle damage and enhance antioxidant responses. The evidence collectively highlights sulforaphane as a promising candidate for preserving skeletal muscle integrity in T2D. However, more randomized controlled trials are required to define optimal dosing, long-term safety, and direct efficacy in patients with sarcopenia and diabetes. This review strengthens the translational value of sulforaphane while integrating mechanistic insights and therapeutic potential against T2D-associated sarcopenia.

## 1. Introduction

Type 2 diabetes (T2D) is a chronic metabolic disorder characterized by insulin resistance and relative insulin deficiency, resulting in persistent hyperglycemia [1]. Clinically, T2D presents symptoms such as polyuria, polydipsia, polyphagia, and unexplained weight loss. Over time, chronic hyperglycemia can lead to microvascular and macrovascular complications, including retinopathy, nephropathy, neuropathy, and cardiovascular diseases [2]. The disease is frequently associated with dyslipidemia and impaired lipid profile, characterized by elevated triglycerides, low-density lipoprotein cholesterol, and reduced high-density lipoprotein cholesterol [3]. Thus, it has become acceptable that effective management of T2D requires comprehensive strategies addressing blood glucose control, lipid profiles, and associated metabolic disturbances [4]. However, uncontrolled diabetes can have a devastating effect within the human body, initiating vast pathological features that encompass muscle wasting, a condition known as sarcopenia.

Sarcopenia, the progressive loss of skeletal muscle mass, strength, and function, is a common and debilitating condition in individuals with T2D [5]. This muscle degradation is exacerbated by the metabolic disturbances inherent in T2D, including insulin resistance, chronic inflammation, and dyslipidemia [6]. Mechanistically, insulin resistance impairs muscle protein synthesis and enhances muscle protein breakdown, while chronic inflammation, driven by elevated levels of pro-inflammatory cytokines, further accelerates muscle loss [6]. Within this pathological state, dyslipidemia contributes to muscle damage through oxidative stress and lipid accumulation, compounding the effects of sarcopenia [6]. The combined impact of these factors leads to frailty, increased risk of falls, and reduced quality of life in affected individuals with T2D [6]. There has been an interest in understanding the pathological consequences, especially the involvement of both oxidative stress and inflammation, in driving muscle wasting in T2D.

Oxidative stress defines exacerbated production of free radicals, mainly reactive oxygen species (ROS), that subsequently deplete the intracellular antioxidant defence systems, leading to enhanced cellular damage [7]. Indeed, oxidative stress plays a critical role in the pathogenesis of sarcopenia, particularly in the context of T2D [8]. During this state, dyslipidemia and chronic hyperglycemia exacerbate the generation of toxic ROS levels that accelerate apoptosis and cellular damage within the skeletal muscle [9]. Even worse, sustained oxidative stress seen in older individuals with diabetes [10] can lead to the damaging effects linked to inflammation. Here, it is proposed that a chronic inflammatory state, marked by increased levels of pro-inflammatory cytokines such as tumor necrosis factor (TNF)-α and interleukin (IL)-6, amplifies muscle protein breakdown and impairs muscle regeneration [11]. Together, both oxidative stress and inflammation create a vicious cycle that accelerates muscle wasting and functional decline in individuals with T2D [6]. This drives the growing interest in discovering the potential therapies that could protect against sarcopenia by neutralizing oxidative stress and inflammation in T2D [12,13,14]. In fact, sulforaphane, an isothiocyanate naturally occurring in high concentrations in cruciferous vegetables, is increasingly studied for its potential therapeutic benefits [15,16,17,18,19,20,21,22].

The reviewed literature confirms the growing interest in sulforaphane as a bioactive compound capable of modulating oxidative stress, inflammation, and mitochondrial dysfunction relevant to skeletal muscle health. For example, Vargas-Mendoza and coworkers hypothesized that phytochemicals like sulforaphane and curcumin could prevent muscle atrophy, albeit their findings were largely based on a nutrigenomic and general muscle health perspective without focusing on sarcopenia in T2D [23]. Similarly, Lilley and colleagues have reviewed evidence, clarifying that broccoli-rich sulforaphane remains a feasible therapeutic target to alleviate mitochondrial myopathies related to sarcopenia, but did not focus on diabetes-associated muscle wasting [24]. Our recent review comprehensively examined evidence on sulforaphane as a nutraceutical to potentially alleviate diabetes-related complications, also without focusing on sarcopenia [25]. The hypothesis driving potential therapeutic effects of sulforaphane predominantly rely on its capacity to activate the nuclear factor erythroid 2-related factor 2 (Nrf2) to protect against diabetes-associated complications [25]. Nevertheless, skeletal muscle outcomes in these analyses are largely limited to insulin sensitivity rather than sarcopenia-specific endpoints such as muscle mass, strength, or function. Additional mechanistic reviews by Kasai et al. [26] and Islam [27] further implicate Nrf2 activation, together with mitochondrial ROS elimination and immunometabolic regulation, as central to insulin resistance and muscle adaptation as part of the therapeutic effects of sulforaphane within the pathological sphere of diabetes. Notably, these insights remain fragmented and are not integrated within a sarcopenia-focused T2D framework. Accordingly, a clear gap remains for an integrative evaluation of sulforaphane as a therapeutic strategy targeting sarcopenia in T2D.

Importantly, no previous review has specifically integrated mechanistic and therapeutic evidence linking sulforaphane to skeletal muscle preservation in the context of T2D-associated sarcopenia. The present review addresses this gap by critically synthesizing evidence from both preclinical and clinical studies on the potential therapeutic effects of sulforaphane against oxidative stress and inflammation to promote skeletal muscle preservation under diabetic conditions. Here, the major component of the reviewed information covers the relevance of physicochemical properties and biological properties of sulforaphane to highlight its potential therapeutic effects.

## 2. An Overview of the Physicochemical and Biological Properties of Sulforaphane

Sulforaphane (Figure 1) is a bioactive compound found predominantly in cruciferous vegetables, such as broccoli, Brussels sprouts, and kale [28]. As such, broccoli sprouts contain significantly higher concentrations of sulforaphane precursors than its mature form. This is supported by early evidence showing that three-day-old broccoli sprouts contain more glucoraphanin than mature broccoli (16.6 μmol/g vs. 1.08 μmol/g, respectively), which translates to a 15-fold increase in glucoraphanin content between these two forms of broccoli [29]. Glucoraphanin is hydrolysed to active sulforaphane via enzymatic action by myrosinase [28]. This enzymatic activity is thermolabile, and the intensity of heat used does significantly affect the amount of sulforaphane eventually produced. In a human kinetic study, raw consumption of broccoli led to a significantly higher bioavailability of sulforaphane (37%) compared to cooked broccoli (3.4%); also, the compound was apparently absorbed more quickly when consumed raw [30]. In this regard, the loss of bioavailability may be offset by the co-administering of exogenous myrosinase from other plant sources, as shown in a randomized crossover trial where myrosinase from mustard seeds almost doubled the bioavailability of sulforaphane (18% to 39%) when compared to glucoraphanin alone [31]. Also, in the event that the myrosinase from dietary sources is denatured during the cooking process, glucoraphanin may be partially transformed into sulforaphane and related compounds by the gut microbiota; however, the extent of transformation varies significantly between individuals and it may be complex to predict the anticipated yield due to the end product being chemically unstable; therefore, intact plant myrosinase-mediated hydrolysis remains superior for yielding higher amounts of the bioactive [32].

Beyond the myrosinase activity and processing methods, the food matrix in which glucoraphanin or sulforaphane is ingested greatly affects its bioavailability. In essence, this matrix is progressively influenced by thermal processing, mastication, industrial operations, and subsequent gastrointestinal digestion [33]. In a human crossover study, the addition of broccoli sprouts to protein, fiber, and lipid gels all significantly decreased the bioavailability of preformed iberin relative to gels that contained no broccoli sprouts, whereas the same decrease in bioavailability for sulforaphane did not reach statistical significance [34]. The dietary cofactors that are co-administered with glucoraphanin can also influence its conversion to sulforaphane. Zhu et al. (2025) showed that the conversion of glucoraphanin to sulforaphane was improved, and bioavailability enhanced, by adding ascorbic acid, a myrosinase cofactor to an in vitro gastrointestinal digestion model [35]. The doses of sulforaphane that have been tested amongst clinical studies range from 9.9 to 847 µmol per day taken orally, with doses of glucoraphanin ranging from 25 to 800 µmol per day [36]. Thus, while it is evident from this review that the bioavailability of sulforaphane from dietary sources is superior to highly processed supplements containing this bioactive, it is not yet fully understood how its dose relates to its efficacy, and what minimum effective dose produces beneficial effects. Consequently, these findings help to suggest that dietary sources of sulforaphane may be more beneficial than supplemental sources of this phytochemical—a factor that must be considered in relation to the desired dietary recommendations for adults with sarcopenia and T2D. Beyond these factors that relate to the introduction of sulforaphane into the system, this broccoli extract exhibits various biological effects within the human body, which relate to the pathophysiology of both sarcopenia and T2D.

Sulforaphane is characterized by its potent antioxidant, anti-inflammatory, and cytoprotective properties [28]. Chemically, it is a thiol-reactive isothiocyanate with the molecular formula C_6_H_11_NOS_2_ [28,37]. The compound exhibits good stability and solubility in aqueous solutions. Pharmacokinetically, sulforaphane is rapidly absorbed in the gastrointestinal tract and distributed throughout the body, where it is metabolized primarily in the liver [38]. Biological studies have demonstrated that sulforaphane activates the Nrf2 pathway [39], a key regulator of antioxidant responses and cellular defence mechanisms. Notably, through this well-studied intracellular mechanism, sulforaphane protects against oxidative stress and inflammation, making it a promising candidate for therapeutic interventions in conditions like sarcopenia and T2D [40,41,42]. To contextualize these properties, key physicochemical and pharmacokinetic characteristics of sulforaphane, as reported in the literature, are summarized in Table 1. This overview provides a framework for understanding its bioavailability, metabolism, and biological relevance in metabolic and muscle-related disorders.

### Direct Molecular Targets and Target Engagement of Sulforaphane

Beyond nonspecific antioxidant effects, sulforaphane acts as an electrophilic signaling molecule that can modify reactive cysteine residues within Kelch-like ECH-associated protein 1 (Keap1). Cys151 is among the well-characterized sensor residues modified following sulforaphane exposure, disrupting Keap1-dependent targeting of Nrf2 for ubiquitination and degradation and thereby facilitating Nrf2 stabilization and downstream antioxidant-response-element signaling [44]. Target engagement is concentration- and time-dependent; however, quantitative information regarding Keap1 cysteine occupancy, persistence of modification and binding kinetics in living skeletal-muscle cells remains limited [45]. This uncertainty is important when translating mechanistic observations from purified proteins or cultured cells to clinically achievable sulforaphane exposures.

Sulforaphane and its metabolites can additionally modulate histone deacetylase activity, providing a potential Nrf2-independent epigenetic mechanism [46,47]. In skeletal muscle, Yang et al. demonstrated involvement of the HDAC8–PGC-1α axis in sulforaphane-induced mitochondrial biogenesis and energy metabolism [20]. Nevertheless, HDAC-mediated effects should currently be regarded as complementary rather than primary skeletal-muscle mechanisms because the concentrations required for direct HDAC inhibition and their relationship to achievable human tissue exposure remain incompletely established. Thus, Keap1–Nrf2 represents a well-characterized direct molecular target pathway, while HDAC regulation provides a potentially important secondary mechanism requiring further translational validation.

## 3. Nrf2 Signaling as an Integrative Regulator of Oxidative Stress and Inflammation in Sarcopenia and T2D

The Nrf2 pathway is widely recognized as a central regulator of cellular defence mechanisms that preserve redox balance and limit stress-induced tissue damage [48]. Under basal conditions, Nrf2 is tightly controlled by Keap1, which promotes its ubiquitination and proteasomal degradation. In response to oxidative or electrophilic stress, Nrf2 escapes Keap1-mediated repression, translocates to the nucleus, and initiates the transcription of antioxidant and cytoprotective genes through binding to antioxidant response elements (AREs) [49]. This adaptive response supports the detoxification of ROS, maintenance of mitochondrial integrity, and protection against macromolecular damage [7,26]. Figure 2 gives an overview of dietary sources, bioconversion, and proposed skeletal-muscle actions of sulforaphane.

In skeletal muscle, Nrf2 signaling is particularly relevant due to the high metabolic demand and continuous ROS generation associated with contractile activity [50]. Importantly, impaired Nrf2 activity has been implicated in muscle vulnerability under pathological conditions characterized by chronic oxidative stress, including aging, insulin resistance, and T2D [8,9,11,12,13]. Regarding the latter, persistent hyperglycemia and dyslipidemia exacerbate mitochondrial ROS production and overwhelm endogenous antioxidant defences, thereby accelerating muscle protein degradation and impairing regenerative capacity [8,9,11,12,13]. Experimental evidence further suggests that reduced Nrf2 responsiveness in insulin-resistant tissues contributes to the progression of muscle dysfunction and progression of sarcopenia [7,26]. Beyond its canonical antioxidant role, Nrf2 is increasingly recognized as a key modulator of inflammatory signaling, a process highly relevant to sarcopenia in T2D [51]. Chronic low-grade inflammation, consistent with raised levels of pro-inflammatory cytokines like TNF-α and IL-6, promotes muscle catabolism and disrupts myogenic differentiation [8,9,11,12,13]. Collectively, the activation of Nrf2 has been shown to suppress inflammatory pathways within a pathological state of T2D; this extends to suppressing the destructive consequences of nuclear factor-kappa B (NF-κB) signaling, thereby limiting cytokine-driven muscle damage [7,26]. This dual antioxidant and anti-inflammatory function positions Nrf2 as an integrative regulator of muscle homeostasis under metabolic stress.

Accumulating preclinical evidence indicates that sulforaphane-mediated Nrf2 activation improves redox balance, attenuates inflammatory signaling, and supports mitochondrial function in skeletal muscle exposed to metabolic, inflammatory, or aging-related stressors [15,16,17,18,19,20,21,22]. These mechanistic actions are particularly relevant in the context of T2D, where oxidative stress and inflammation converge to accelerate muscle wasting [8]. Collectively, available preclinical evidence establishes Nrf2 as a critical mechanistic link between oxidative stress, inflammation, and metabolic dysfunction in sarcopenia associated with T2D [15,16,17,18,19,20,21,22]. Thus, sulforaphane offers a biologically plausible strategy to counteract multiple drivers of muscle deterioration by targeting this pathway. These insights provide a mechanistic basis to explore sulforaphane’s potential therapeutic effects on skeletal muscle, which are subsequently examined in experimental and clinical studies.

## 4. Evidence on the Potential Therapeutic Effects of Sulforaphane Against Sarcopenia in T2D

Here, a comprehensive approach was applied to search, identify, and synthesize evidence best informing on the potential therapeutic effects of sulforaphane against sarcopenia in T2D. Briefly, a literature search through PubMed was conducted using the terms *“sulforaphane and sarcopenia”*, *“sulforaphane and type 2 diabetes”*, and combinations such as *“muscle wasting and type 2 diabetes”*, yielding a total of 52 records for initial consideration [52]. These records were subsequently filtered and eventually limited to original studies evaluating sulforaphane or sulforaphane-related interventions and reporting outcomes directly relevant to skeletal muscle health, muscle wasting, sarcopenia, or metabolic dysfunction affecting skeletal muscle. To broaden the scope of study inclusion, additional searches were performed in other databases such as the Cochrane Library, Scopus, and Google Scholar, in accordance with approaches suggested by previous reviews [53,54]. These searches were conducted without restriction on publication dates, up to December 2025. Reviews and other secondary literature were used to provide background and mechanistic context but were not included in the evidence tables. Two independent reviewers screened the potentially relevant studies and extracted relevant data, which were summarized in table format for each article. This included the experimental model, dose and duration of treatment, and main findings related to the potential therapeutic effects of sulforaphane on sarcopenia. Following relevance screening and removal of overlapping reports, 19 unique primary studies are summarized across subsequent sections. Because this study was designed as a narrative rather than a systematic review, no formal risk-of-bias or certainty-of-evidence grading was undertaken. The subsequent sections synthesize this evidence, highlighting mechanistic insights from in vitro and in vivo models as well as emerging clinical data, with particular emphasis on the modulation of oxidative stress and inflammation, potentially via Nrf2 activation and any other related mechanisms.

### 4.1. An Overview of Characteristic Features of Included Studies

Available evidence evaluating sulforaphane as a potential therapeutic candidate for sarcopenia spans in vitro skeletal-muscle models and in vivo models of metabolic stress and aging. In fact, preclinical investigations predominantly employed C2C12 myoblasts or myotubes exposed to metabolic, inflammatory, or contractile stressors to mimic muscle wasting conditions (Table 2). Likewise, animal studies focused on aging, diet-induced obesity, diabetes, and muscle injury models. Notably, across experimental studies, sulforaphane doses and exposure durations varied substantially, ranging from short-term cellular treatments (minutes to hours) to chronic dietary supplementation in rodents lasting up to 20 weeks (Table 3). Mechanistically, most studies converged on the activation of Nrf2 signaling and downstream antioxidant pathways as central mediators of sulforaphane’s protective effects on skeletal muscle (Table 2 and Table 3). In contrast, clinical evidence remains sparse and largely restricted to short-term intervention studies in healthy individuals, with no randomized controlled trials directly evaluating sulforaphane in individuals with sarcopenia and T2D (Table 4). Overall, the included studies were judged to be of moderate-to-high quality, with most experimental studies employing appropriate controls and mechanistic validation.

### 4.2. Evidence of Sulforaphane Against Sarcopenia in Cultured Skeletal Muscles Subjected to Metabolic Stress

Cultured skeletal muscle models have provided foundational mechanistic insight into the protective effects of sulforaphane against sarcopenia-related stressors [16,17]. Most studies employed C2C12 myoblasts or differentiated myotubes subjected to metabolic, inflammatory, or oxidative insults, enabling direct assessment of intracellular antioxidants and myogenic responses (Table 2). For example, early work by Whitman et al. (2013) demonstrated that short-term exposure to sulforaphane (2 µM for 4 h) robustly activated Nrf2 signaling, leading to enhanced mitochondrial subunit expression and restoration of myosin isoform profiles associated with improved muscle contractile capacity [15]. Subsequent studies consistently reported reductions in intracellular ROS and protein oxidation following sulforaphane treatment. For instance, Moon et al. (2020) showed that 5 µM sulforaphane reversed serum starvation-induced muscle atrophy by activating the Nrf2/heme oxygenase one (HO-1) pathway and suppressing oxidative damage [17].

Consistent with the ameliorative effects against oxidative stress-associated cellular damage, the anti-inflammatory properties were also evident in models of lipid- and endotoxin-induced stress. This was shown by Faridvand et al. (2020), reporting that sulforaphane (750 µM, 24 h) significantly reduced palmitate-induced IL-6 and TNF-α expression while enhancing Nrf2 and uncoupling protein 3 (UCP3) levels [16]. Similarly, Wang et al. (2020) demonstrated that sulforaphane (1–30 µM) attenuated lipopolysaccharide-induced inflammation by inhibiting Toll-like receptor-4 (TLR4) and NLRP3 inflammasome signaling, thereby promoting myogenic differentiation [18].

Several studies further highlighted sulforaphane’s capacity to improve mitochondrial function. Yang et al. (2023) reported enhanced mitochondrial biogenesis and ATP production following brief sulforaphane exposure via upregulation of peroxisome proliferator-activated receptor-γ coactivator-1α (PGC1α) [20], findings corroborated by studies using electrically stimulated myotubes, which demonstrated improved mitochondrial respiration, antioxidant enzyme expression, and adenosine triphosphate (ATP) synthesis [21,22].

Notably, not all studies evaluated sarcopenia-specific outcomes. Luo et al. (2023) reported improved glucose uptake following low-dose sulforaphane exposure without assessing oxidative stress or mitochondrial endpoints [19]. Additionally, findings from non-muscle cell models limit direct extrapolation to skeletal muscle physiology. Collectively, these in vitro studies strongly support sulforaphane-mediated activation of Nrf2 signaling as a central mechanism protecting skeletal muscle against oxidative and inflammatory stress, beyond its capacity to improve mitochondrial function (Table 2). Notably, the observed effects varied in terms of dose and duration, consistent with the application of diverse experimental models.

### 4.3. Evidence of Sulforaphane Against Sarcopenia in Preclinical Models of T2D and Related Complications

Preclinical studies using cultured skeletal muscle models have provided key information on the potential therapeutic effects of sulforaphane against sarcopenia-related stress conditions. Indeed, several experimental setups have demonstrated positive outcomes, with sulforaphane effectively modulating oxidative stress, inflammation, and mitochondrial function [17,22]. Beyond this, rodent models have extended in vitro findings by demonstrating the systemic and functional effects of sulforaphane on skeletal muscle under conditions of aging, metabolic stress, and diabetes (Table 3). These studies support the therapeutic potential of sulforaphane in improving muscle integrity and function primarily through modulation of oxidative stress, inflammation, and mitochondrial homeostasis. For example, in early work, Malaguti et al. (2009) showed that short-term sulforaphane administration (25 mg/kg for 3 days) enhanced antioxidant enzyme activity and reduced markers of muscle damage following exhaustive exercise in rats [55]. Aging and metabolic disease models further affirmed the relevance of Nrf2 signaling within the studied therapeutic potential of sulforaphane. Here, Bose et al. (2020) demonstrated that dietary sulforaphane restored age-associated declines in Nrf2 activity, mitochondrial function, and exercise capacity in aged mice [56]. In type 2 diabetic (*db*/*db*) mice, sulforaphane improved skeletal muscle function by attenuating oxidative stress, inflammation, and apoptotic signaling via Nrf2/HO-1 activation [57]. Additional studies identified complementary mechanisms, including improved mitochondrial dynamics and lipid metabolism. Yan et al. (2021) showed that sulforaphane stabilized dynamin-related protein-1 (Drp1), reducing mitochondrial dysfunction within the skeletal muscle of aged mice [58], while Zhang et al. (2025) reported enhanced muscle fiber integrity through regulation of AMP-activated protein kinase (AMPK) signaling and lipid oxidation [62]. However, variability in dosing regimens, treatment duration, and outcome measures complicates direct comparison across studies. To facilitate translational interpretation, body-surface-area-normalized human equivalent dose (HED) estimates are therefore provided in Table 3 for studies reporting sulforaphane doses relative to animal body weight; dietary concentrations were not converted where actual body-weight-normalized exposure could not be reliably determined [63]. Moreover, some investigations reported metabolic benefits without substantial effects on body composition or food intake [19]. Overall, in vivo evidence strongly supports sulforaphane’s capacity to preserve skeletal muscle health under diabetic and aging-related stress, primarily through antioxidant and mitochondrial modulating mechanisms.

### 4.4. Clinical Evidence of Sulforaphane Against Sarcopenia in T2D

Clinical studies are essential for evaluating the pharmacological effects of bioactive compounds, including sulforaphane, against sarcopenia in T2D. These studies are pivotal in translating findings from preclinical models to human applications, ensuring that the observed benefits and mechanisms apply to patients [66]. Clinical evidence evaluating sulforaphane in the context of muscle health remains limited and largely exploratory (Table 4). To date, the available studies have been conducted exclusively in healthy young adults and have primarily focused on exercise-induced muscle damage rather than sarcopenia or T2D. For instance, Komine et al. (2022) demonstrated that short-term sulforaphane supplementation (30 mg/day for 2 weeks) reduced delayed-onset muscle soreness following exercise, accompanied by increased expression of NQO1 and reduced lipid peroxidation [18]. Similarly, Sato et al. (2021) reported that sulforaphane supplementation suppressed exercise-induced increases in inflammatory cytokines, particularly IL-6, suggesting an anti-inflammatory effect in skeletal muscle [65]. Although these findings provide proof-of-concept that sulforaphane can modulate some oxidative stress and inflammatory responses in human muscle, no studies have directly evaluated its effects on muscle mass, strength, or functional outcomes in individuals with sarcopenia or T2D. This represents a critical translational gap that warrants well-designed randomized controlled trials in clinically relevant populations. Table 5 provides a concise comparison of sulforaphane dosing and observed outcomes across in vitro, rodent, and clinical models. Notably, HED estimates vary substantially across preclinical models, with some body-weight-normalized exposures overlapping plausible human exposure ranges, while others remain considerably higher; differences in formulation, dosing frequency, bioavailability, and pharmacokinetics nevertheless limit direct dose equivalence between experimental and clinical studies. This table highlights the translational gap between mechanistic findings in experimental models and the limited clinical evidence currently available.

## 5. Summary and Future Perspectives

Sarcopenia represents a critical complication of T2D, exacerbated by the combined effects of aging, insulin resistance, chronic inflammation, and oxidative stress [67]. These pathological factors converge to impair skeletal muscle mass, strength, and function, ultimately diminishing the quality of life in affected individuals [5,6]. Central to this pathological process is the dysregulation of intracellular antioxidant defences and inflammatory pathways, with Nrf2 emerging as a pivotal regulator. Notably, Nrf2 serves as a unifying mechanistic hub connecting oxidative stress, mitochondrial dysfunction, and muscle wasting by coordinating transcriptional programs that maintain redox homeostasis and suppress pro-inflammatory signaling [7,26,51]. Consequently, pharmacological or dietary activation of Nrf2 represents a compelling strategy to counteract sarcopenia in the context of T2D.

The evidence synthesized in this review indicates that sulforaphane activates Nrf2 and related cytoprotective pathways across several experimental skeletal-muscle models, mitigating oxidative damage and inflammation across in vitro and in vivo settings (Table 2 and Table 3). In cultured skeletal-muscle models, sulforaphane consistently reduced ROS, restored mitochondrial integrity via PGC1α signaling, and suppressed pro-inflammatory mediators, including TNF-α, IL-6, and additional chemokines [15,16,17,18,19,20,21,22]. Preclinical rodent studies further corroborated these findings, revealing improved muscle mass, contractile function, and metabolic homeostasis, often linked to AMPK activation, UCP3 upregulation, and enhanced mitochondrial dynamics [56,57,58,59,61,62]. Importantly, these mechanistic effects were observed across diverse models, ranging from high-fat diet-induced obesity and T2D to age-related muscle decline. Despite compelling preclinical evidence (Table 2 and Table 3), clinical studies remain limited and largely confined to healthy young adults undergoing exercise interventions [64,65]. The two included trials demonstrated reductions in muscle damage markers and improved antioxidant gene expression with sulforaphane intake, albeit the direct impact of this isothiocyanate on sarcopenia in T2D remains to be established. Pharmacokinetic data indicates rapid absorption, broad tissue distribution, including in skeletal muscle, and metabolism via the mercapturic acid pathway, highlighting both bioavailability and potential dosing considerations for therapeutic use [38,48,49,50,51].

In summary, our findings highlight the therapeutic potential of sulforaphane as a multi-target intervention for sarcopenia in T2D, primarily through Nrf2-mediated antioxidant and anti-inflammatory pathways (Figure 3). This is consistent with its augmented effect by ancillary mechanisms involving AMPK, UCP3, and mitochondrial regulation. Nevertheless, heterogeneity in experimental designs, dosing regimens, and outcome measures, as well as the paucity of clinical evidence, limit direct translational conclusions. In fact, the marked discrepancy between doses used in rodent models and those evaluated in humans highlights a major translational challenge. Future clinical trials should investigate dose optimization, formulation strategies that improve bioavailability, and long-term safety in patients with T2D and sarcopenia. This lays a foundation for future research to prioritize randomized controlled trials in T2D patients with sarcopenia to explore optimal dosing, long-term safety, and functional outcomes. Ultimately, sulforaphane offers a promising avenue to restore muscle integrity, ameliorate metabolic dysfunction, and enhance the quality of life in individuals burdened by T2D-associated sarcopenia.

## Figures and Tables

**Figure 1:**
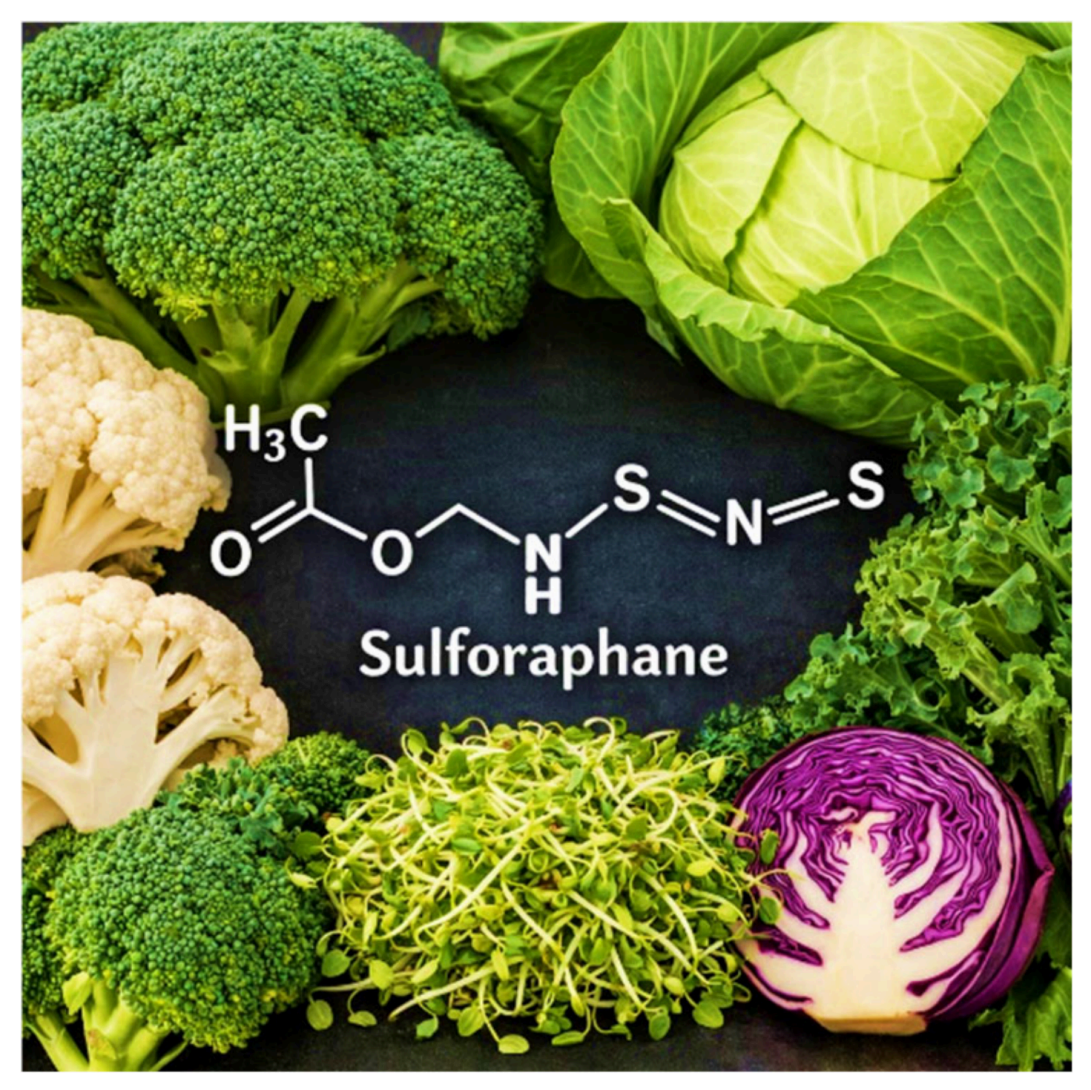
Sulforaphane is abundant in cruciferous vegetables. Sulforaphane, a potent antioxidant and nuclear factor erythroid 2-related factor 2 (Nrf2) activator, is present in high concentrations in cruciferous vegetables such as broccoli, cauliflower, Brussels sprouts, and kale. Briefly, upon ingestion, sulforaphane is absorbed in the gastrointestinal tract before it enters skeletal muscle cells and potentially activates Nrf2 by covalently modifying Kelch-like ECH-associated protein 1 (Keap1). This process triggers cellular antioxidant defences, supports mitochondrial health, and reduces oxidative stress to preserve muscle integrity in type 2 diabetes.

**Figure 2:**
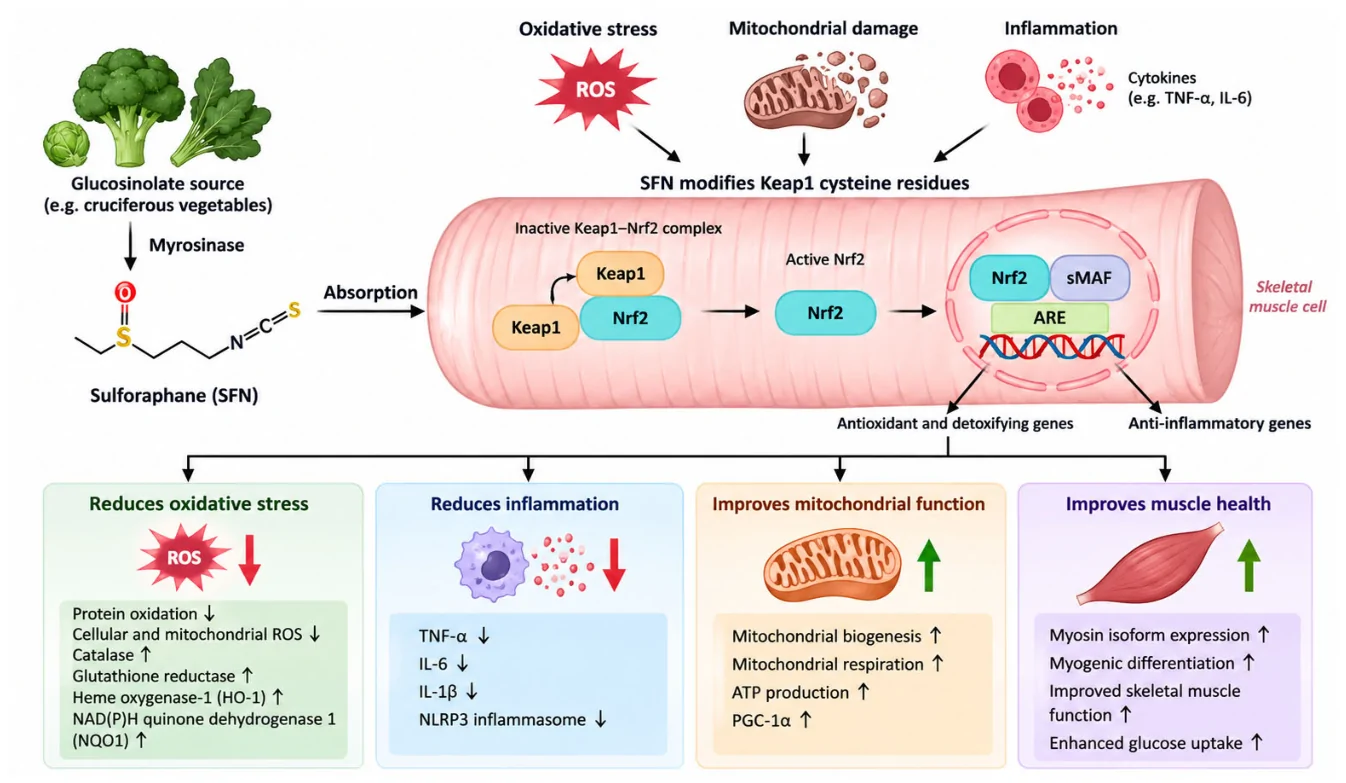
Dietary sources, bioconversion, and proposed skeletal-muscle actions of sulforaphane. Glucoraphanin derived from cruciferous vegetables is converted to sulforaphane (SFN) primarily through myrosinase-mediated hydrolysis. Briefly, following gastrointestinal absorption and systemic distribution, SFN can modify reactive cysteine residues of Kelch-like ECH-associated protein 1 (Keap1), thereby stabilizing nuclear factor erythroid 2-related factor 2 (Nrf2), promoting its nuclear translocation and activation of antioxidant response element (ARE)-dependent cytoprotective signalling. Activation of this pathway is associated with enhanced antioxidant and detoxification responses, attenuation of oxidative stress and inflammatory signaling, improved mitochondrial function, and preservation of skeletal-muscle homeostasis. Upward arrows (↑) indicate increased expression, activity, or function, whereas downward arrows (↓) indicate decreased expression, activity, or function. Abbreviations: ARE, antioxidant response element; ATP, adenosine triphosphate; HO-1, heme oxygenase-1; IL, interleukin; Keap1, Kelch-like ECH-associated protein 1; NLRP3, NOD-, LRR- and pyrin domain-containing protein 3; NQO1, NAD(P)H quinone dehydrogenase 1; Nrf2, nuclear factor erythroid 2-related factor 2; PGC-1α, peroxisome proliferator-activated receptor gamma coactivator 1-alpha; ROS, reactive oxygen species; SFN, sulforaphane; sMAF, small musculoaponeurotic fibrosarcoma proteins; TNF-α, tumor necrosis factor-alpha. The figure was created by the authors with the assistance of OpenAI’s ChatGPT image-generation tool (2026). The scientific content, labels, interpretation, and final figure were reviewed and approved by the authors.

**Figure 3:**
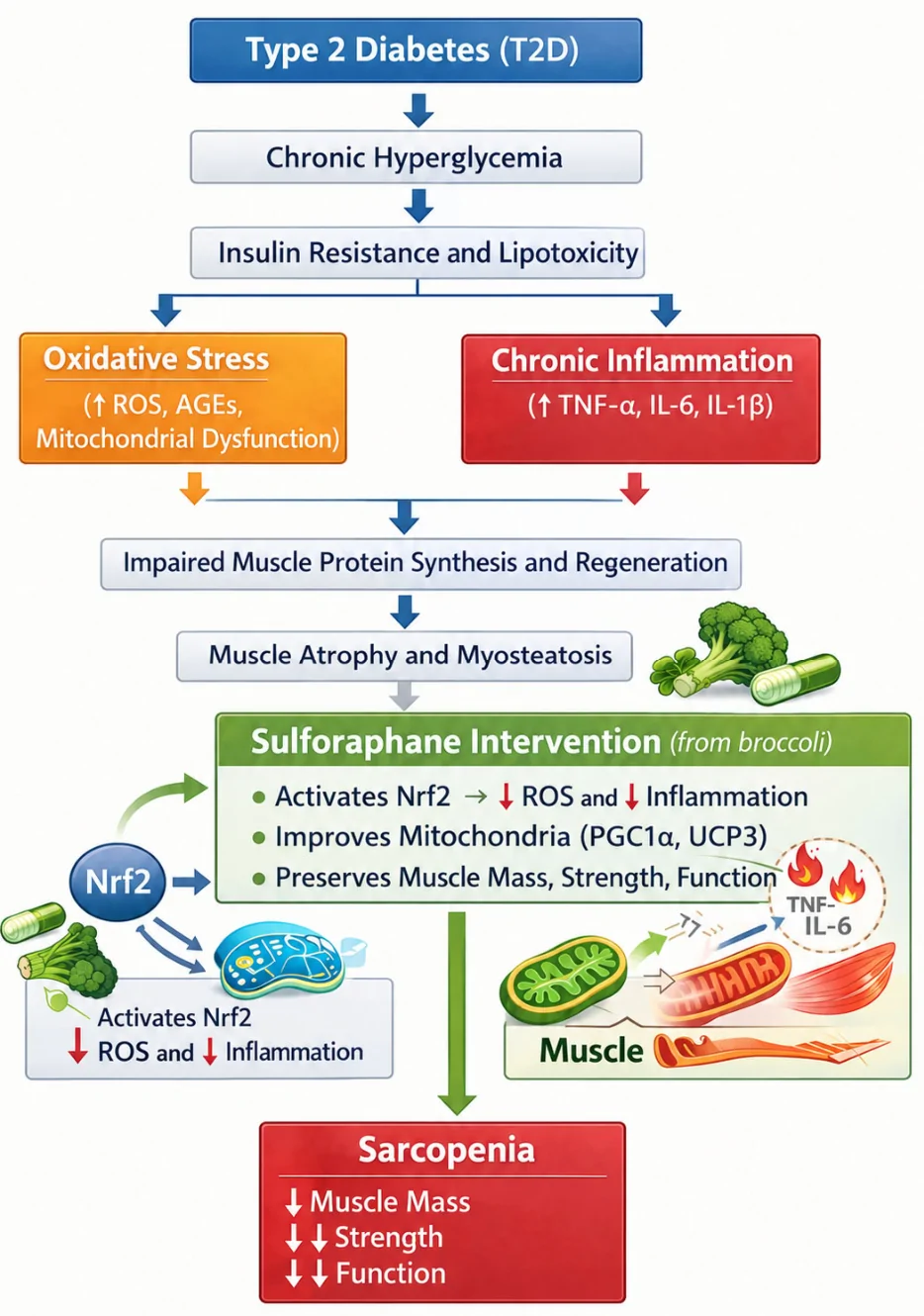
Mechanistic overview of sulforaphane-mediated protection against sarcopenia in type 2 diabetes (T2D). Briefly, chronic hyperglycemia in T2D induces insulin resistance and lipotoxicity, promoting oxidative stress and chronic inflammation, which impair skeletal muscle protein synthesis and regeneration, ultimately causing sarcopenia (muscle loss). Sulforaphane, a bioactive compound found in cruciferous vegetables, activates the nuclear factor erythroid 2-related factor 2 (Nrf2) pathway by modifying Kelch-like ECH-associated protein 1 (Keap1). This activation reduces reactive oxygen species (ROS), advanced glycation end-products (AGEs), and inflammatory cytokines [tumor necrosis factor-alpha (TNF-α), interleukin-6 (IL-6), interleukin-1 beta (IL-1β)], while improving mitochondrial function via peroxisome proliferator-activated receptor gamma coactivator 1-alpha (PGC1α) and uncoupling protein 3 (UCP3). Together, these effects preserve muscle mass, strength, and function. Arrow, regardless of color, indicators: an arrow facing up indicates increased expression/activation; the arrow facing down alternatively demonstrates reduced expression/deactivation.

**Table 1:** Physicochemical and pharmacokinetic properties of sulforaphane reported in the literature.

Property (Units)	Value/Description	Reference
Chemical class	Isothiocyanate	[27]
Chemical structure	1-Isothiocyanato-4-(methylsulfinyl)butane	[27]
Molecular formula	C_6_H_11_NOS_2_	[27]
Molecular weight (g/mol)	177.29	[27,28]
Source compound	Derived from glucoraphanin via myrosinase hydrolysis	[27]
Physical state	yellow and oily substance	[43]
Aqueous solubility	Soluble in water; acetonitrile, methanol, ethanol, ethyl acetate, dichloromethane, chloroform	[43]
Log P (predicted)	~1.0–1.5 (moderate lipophilicity)	[28]
Gastrointestinal absorption	Rapid oral absorption	[28,33]
Bioavailability	High, dependent on myrosinase activity and gut microbiota	[33,34]
Plasma Tmax	~1–3 h post-ingestion	[28,33]
Primary metabolism	Mercapturic acid pathway (glutathione conjugation)	[28,35]
Major metabolites	Sulforaphane-GSH, -CysGly, -Cys, -NAC conjugates	[35,36]
Tissue distribution	Liver, skeletal muscle, adipose tissue	[28,36]
Elimination route	Renal (urinary excretion of metabolites)	[35]
Biological half-life	~2–3 h (metabolite-dependent)	[33,35]
Primary molecular target	Keap1 cysteine residues	[29,30]
Key biological actions	Nrf2 activation, antioxidant, and anti-inflammatory effects	[29,30,31,32]

**Table 2:** Potential therapeutic effects of sulforaphane against sarcopenia in experimental models of cultured skeletal muscles subjected to conditions of stress.

Author, Year	Country	Experimental Model	Dose and Duration of Treatment	Main Findings
Whitman et al. (2013) [15]	United States	Skeletal muscle cells (C2C12) myoblasts were allowed to differentiate	Received sulforaphane at 2 μM for 4 h before harvest	Enhanced intracellular antioxidant response, including muscle strength, by modulating nuclear factor erythroid 2-related factor 2 (Nrf2) activity. Reversed alterations in myosin isoform expression and improved mitochondrial subunit expression through the regulation of peroxisome proliferator-activated receptor gamma coactivator 1 alpha (PGC1α).
Faridvand et al. (2020) [16]	Iran	C2C12 myotubes, subjected to palmitic acid	Received sulforaphane at doses between 1 and 10 μM for 1 h	Alleviated lipid-induced toxicity by ameliorating inflammation, including reducing the gene expression of interleukin six (IL)-6 and tumor necrosis factor-alpha (TNF-α). This was followed by a reduction in reaction levels (ROS levels) and enhanced expression of Nrf2 and uncoupling protein three (UCP3).
Moon et al. (2020) [17]	South Korea	C2C12 myotubes, subjected to serum starvation-induced muscle atrophy	Received sulforaphane at 5 μM for 3 h before serum starvation or menadione exposure	Reversed pathophysiological consequences of muscle atrophy by reducing intracellular reactive oxygen species (ROS) and protein oxidation in atrophic myotubes. Also, upregulated the Nrf2/heme oxygenase one (HO-1) pathway.
Wang et al. (2020) [18]	China	C2C12 myoblasts subjected to reversed lipopolysaccharide	Received sulforaphane at doses between 1 and 30 μM for 6 to 120 h	Reversed lipopolysaccharide-induced inflammation and enhanced myogenic differentiation by decreasing the levels of IL-1β secretion and intracellular ROS production. This was associated with the inhibition of inflammation by targeting Toll-like receptor four (TLR4) and NOD-, LRR-, and pyrin domain-containing protein 3 (NLRP3) pathways.
Luo et al. (2023) [19]	China	Primary human skeletal muscle cells and C2C12 myotubes allowed to differentiate	Received sulforaphane at doses at 0.1 nM or 1 µM for 1 h	Improved the metabolism of skeletal muscle cells by increasing glucose uptake.
Yang et al. (2023) [20]	South Korea	C2C12 myotubes subjected to selective histone deacetylase 8 (HDAC8) inhibition	Received sulforaphane at doses between 4 and 10 μM for 15 min–24 h	Reversed histone deacetylases induced skeletal muscle damage by improving mitochondrial biogenesis and energy (APT) production by upregulating the protein expression of peroxisome proliferator-activated receptor-γ coactivator-1α (PGC1α).
Champsi and Hood (2024) [21]	Canada	C2C12 myotubes subjected to electrical stimulation	Received sulforaphane at 10 μM for 3–48 h	Improved mitochondrial adaptations by enhancing key antioxidant proteins (catalase and glutathione reductase). Reduced cellular and mitochondrial ROS emission and enhanced mitochondrial biogenesis through PGC1α. Activated Acute stress-kinase signaling (AMPK, JNK1), although temporarily.
Moradi et al. (2025) [22]	Canada	Healthy C2C12 myotubes	Received sulforaphane at 5 μM and 10 μM for 4–48 h	Improved skeletal muscle function by increasing intracellular response and through enhancing Nrf2 nuclear localization. This led to increased levels of antioxidant enzymes like NAD(P)H: quinone oxidoreductase one (NQO1) and HO-1. Mitochondrial respiration and ATP production also improved

**Table 3:** Potential therapeutic effects of sulforaphane against sarcopenia in experimental models involving mice and rats subjected to metabolic stress and senescence.

Author, Year	Country	Experimental Model	Dose and Duration of Treatment	Main Findings	Human Equivalent Dose (HED) mg/kg
Malaguti et al. (2009) [55]	Italy	Adult male Wistar rat model of an acute exhaustive exercise protocol	Received 25 mg/kg sulforaphane 25 for 3 days	Strengthened muscle function by increasing intracellular antioxidant enzyme activity, including NAD(P)H: quinone oxidoreductase one (NQO1), glutathione-S-transferase, and glutathione reductase. Also, alleviated muscle damage by reducing levels of plasma lactate dehydrogenase and creatine phosphokinase and preserved myofibre structure.	4.05
Bose et al. (2020) [56]	United States	21–22-month-old aged C57BL/6 male mice	Received a diet supplemented with 442.5 mg/kg of sulforaphane for 12 weeks	Improved skeletal muscle function by restoring nuclear factor erythroid 2-related factor (Nrf2) activity and enhancing mitochondrial function, exercise capacity, and glucose tolerance, while also promoting skeletal muscle satellite cell activation in older mice. Additionally, sulforaphane counteracted age-related muscle dysfunction and oxidative stress.	Not directly estimable from reported dietary concentration
Wang et al. (2020) [57]	China	Type 2 diabetic (*db*/*db*) mice	Received 0.5 mg/kg sulforaphane for 4 weeks	Reversed skeletal muscle dysfunction by ameliorating oxidative stress, inflammation, and apoptotic markers. This was through the activation of the Nrf2/heme oxygenase (HO)-1 signaling pathway and enhancing lipid oxidation.	0.04
Yan et al. (2021) [58]	China	20–22-month-old male Nrf2 knockout (Nrf2−/−) C57BL/6 J mice lacking the *Nrf2* gene and age-matched wild-type (WT) C57BL/6 J control mice	Received 1 mg/kg sulforaphane times/5 days/week for 1 month	Improved sarcopenia-like symptoms via the activation of Nrf2 signaling and stabilization of dynamin-related protein 1 (Drp1), which facilitated mitochondrial fission and reduced mitochondrial dysfunction.	0.08 per administered dose
Wang et al. (2022) [59]	China	8-week-old male SPF C57BL/6 J mice, radiation-induced muscle fibrosis model	Received 0.5 mg/kg sulforaphane 5 times/week for 4 weeks	Protected against radiation-induced muscle fibrosis by enhancing Nrf2-mediated antioxidant activity and downregulated TGF-β1/Smad pathways.	0.04 per administered dose
Luo et al. (2023) [19]	China	6-week-old, high-fat diet (HFD) young male C57BL/6 mice, 8-month-old, HFD middle-aged male C57BL/6 mice, 8-month-old, middle-aged, HFD until obese male C57BL/6 mice	Received 0.5 g/kg, 0.25 g/kg, 40 mg/kg sulforaphane for 18 weeks, 7 weeks, and up to 30 days	Suppressed fat accumulation while improving insulin sensitivity and blood lipid profiles. However, it had minimal effects on body composition or food.	Not directly estimable from reported dietary concentration
Yang et al. (2023) [20]	South Korea	6–8-week-old C57BL/6 J, HFD-induced obese male mice	Received sulforaphane at 50 mg/kg body weight for 15 weeks	Sulforaphane had positive effects on mitochondrial function, suggesting a protective role against obesity-related metabolic diseases, particularly through HDAC8-PGC1α signaling.	4.05
Ye et al. (2023) [60]	Japan	16-week-old, Type 2 diabetic male (KKAy) mice	Received sulforaphane at 10 mg/kg every 12 h for up to 4 weeks	Improved skeletal-muscle insulin sensitivity by increasing levels of selenoprotein P, a major selenoprotein in serum predominantly produced in the liver. This was independent of Nrf2 activation.	0.81 per administered dose
Guo et al. (2025) [61]	China	3-month-old male senescence (SAMP8) mice with accelerated aging	Received 4 mg/kg sulforaphane for 4 weeks	Alleviated sarcopenia-linked muscle pathology by reducing intestinal fibrosis, oxidative stress, inflammation, apoptosis, and muscle atrophy markers while repairing mitochondrial damage. This was consistent with improved mitochondrial biogenesis, morphology and energy-sensing pathways.	0.32 per administered dose
Zhang et al. (2025) [62]	China	4-week-old healthy C57BL/6 J male mice	Received 1 mg/kg (SFN1) or 3 mg/kg (SFN3) for 8 weeks	Strengthened muscle fibers by alleviating lipid oxidation and improving cellular metabolism. This was partially through effectively regulating AMP-activated protein kinase (AMPK) signaling and cholesterol metabolism.	0.08

Footnote: HED, Human Equivalent Dose. HED was calculated using body-surface-area normalization according to Nair and Jacob (2016) [63]: HED (mg/kg) = animal dose (mg/kg) × (Km_animal/Km_human), using Km values of 3 for mice, 6 for rats, and 37 for humans. For interventions reported as dietary concentrations per kilogram of feed rather than as body-weight-normalized doses, HED was not estimated unless sufficient information was available to derive actual daily exposure. For intermittently or repeatedly administered interventions, HED refers to the corresponding body-weight-normalized dose per administration unless otherwise stated. HED estimates are intended for translational dose comparison and do not account for interspecies differences in route of administration, bioavailability, metabolism, or pharmacokinetics.

**Table 4:** Potential therapeutic effects of sulforaphane against sarcopenia in a clinical setting.

Author, Year	Country	Experimental Model	Dose and Duration of Treatment	Main Findings
Komine et al. (2022) [64]	Japan	Healthy individuals (*n* = 16), 20–22 years	Received 30 mg per 3 tablets (SGS precursor converted to sulforaphane) daily for 2 weeks before exercise and continued for 4 days after exercise	Delayed onset muscle soreness. This was consistent with enhanced expression levels of genes for NAD(P)H: quinone oxidoreductase one (NQO1), and lowered lipid peroxidation by decreasing malonaldehyde content
Sato et al. (2021) [65]	Japan	Healthy individuals (*n* = 10) subjected to exercise, with an average age of 22.2 ± 0.3 years	Received sulforaphane at 30 mg/day for 4 weeks	Suppressed the heavy resistance exercise-induced increase in muscle damage parameter and expression of inflammatory cytokines. This included the reduction in interleukin-6 (IL-6) l, especially from baseline to 30 min after exercise, suggesting an anti-inflammatory effect

**Table 5:** Preclinical vs. clinical dose table (Concise comparison).

Model	Dose and Duration	Main Observations	Reference
In vitro (C2C12 myotubes/myoblasts)	0.1–750 µM, 15 min–24 h	Activated nuclear factor erythroid 2-related factor 2 (Nrf2), reduced reactive oxygen species (ROS), improved mitochondrial function, suppressed inflammation	[15,16,17,18,19,20,21,22]
Rodent models (mice/rats)	0.5–442.5 mg/kg diet or 0.5–25 mg/kg body weight, 3 days–20 weeks	Improved muscle mass, contractility, antioxidant activity, mitochondrial function; attenuated inflammation	[56,57,58,59,61,62]
Clinical studies (healthy adults)	30 mg/day orally, 2–4 weeks	Reduced exercise-induced muscle damage, improved antioxidant gene expression, and decreased inflammatory markers	[64,65]

## Data Availability

No new data were created or analyzed in this study. Data sharing is not applicable to this article.

## Abbreviations

AMPK—AMP-activated protein kinase; ARE—Antioxidant response element; HO-1—Heme oxygenase-1; IL—Interleukin; Nrf2—Nuclear factor erythroid 2-related factor 2; ROS—Reactive oxygen species; T2D—Type 2 diabetes; TNF-α—Tumor necrosis factor-alpha; UCP3—Uncoupling protein 3.
